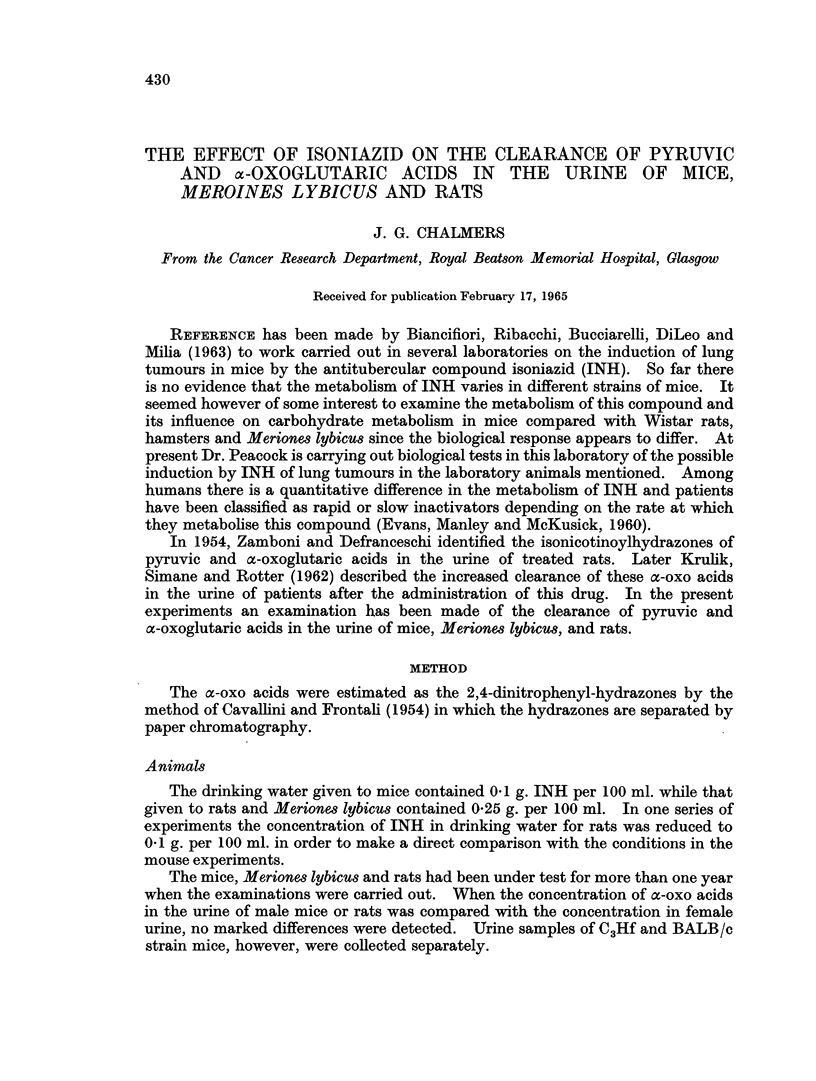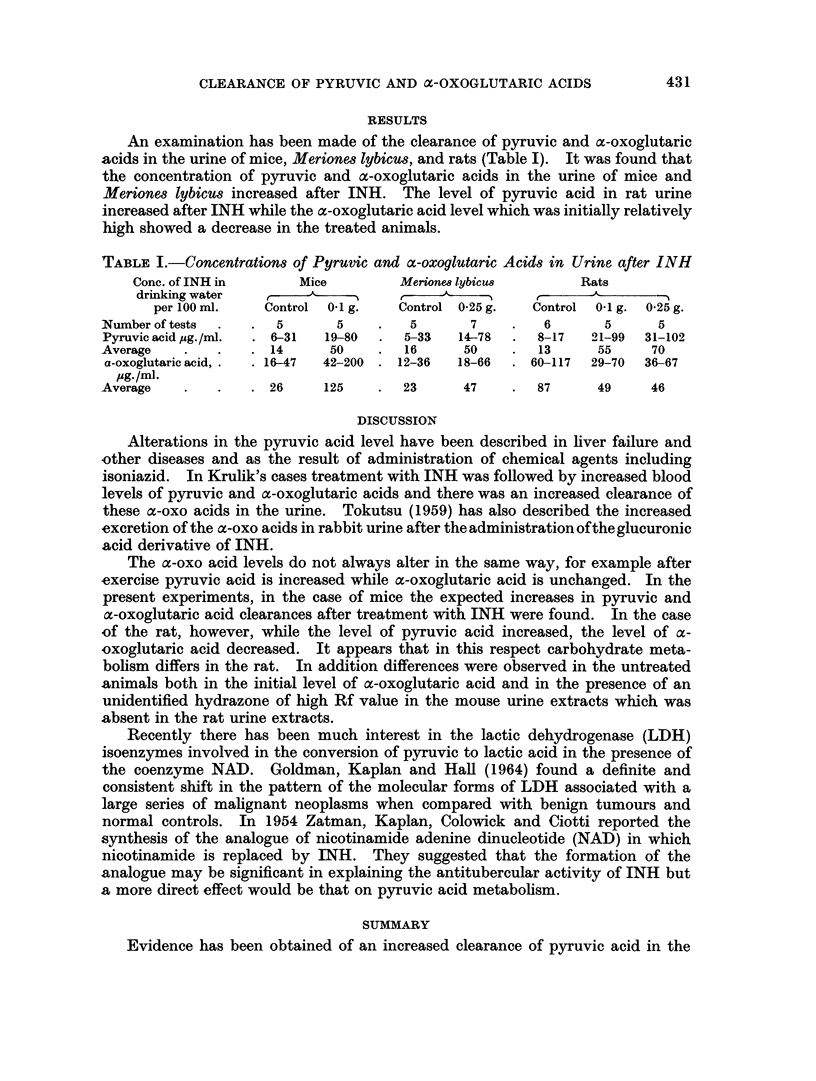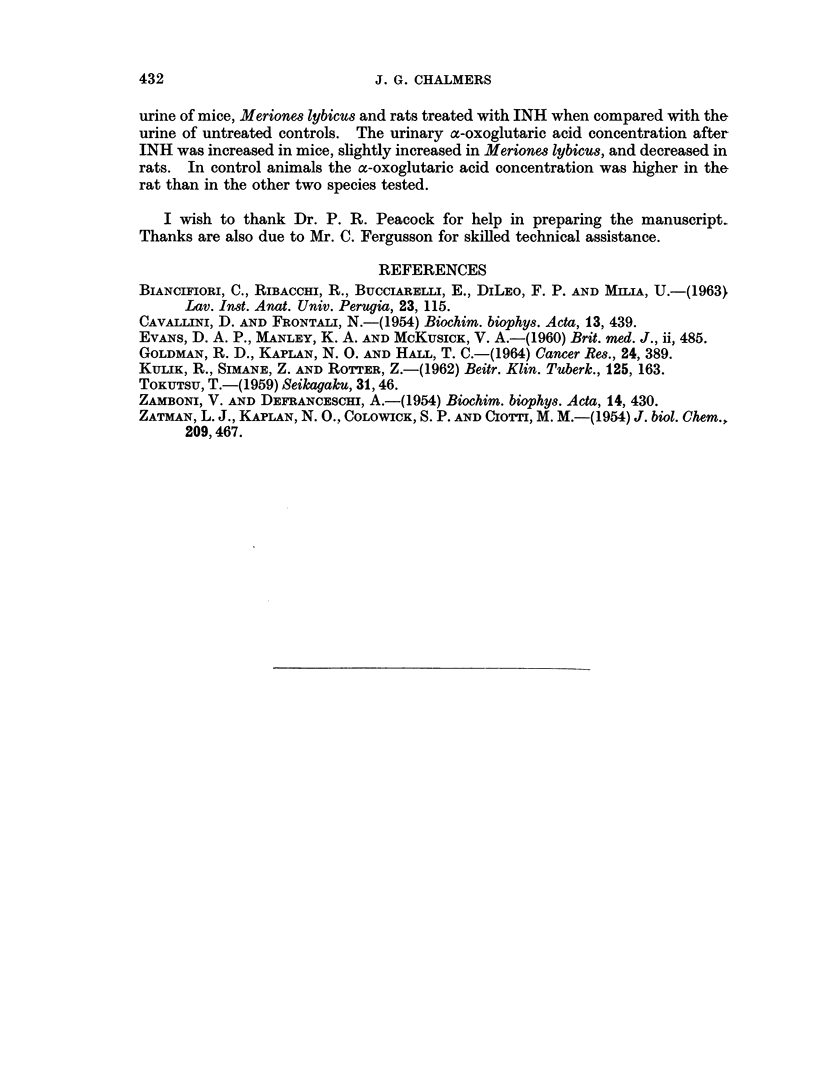# The Effect of Isoniazid on the Clearance of Pyruvic and α-Oxoglutaric Acids in the Urine of Mice, Meriones lybicus and Rats

**DOI:** 10.1038/bjc.1965.51

**Published:** 1965-06

**Authors:** J. G. Chalmers


### THE EFFECT OF ISONIAZID ON THE CLEARANCE OF PYRUVIC AND $\alpha$-OXOGLUTARIC ACIDS IN THE URINE OF MICE, *MEROINES LYBICUS* AND RATS

J. G. CHALMERS

*From the Cancer Research Department, Royal Beatson Memorial Hospital, Glasgow*

Received for publication February 17, 1965

REFERENCE has been made by Biancifiori, Ribacchi, Bucciarelli, DiLeo and Milia (1963) to work carried out in several laboratories on the induction of lung tumours in mice by the antitubercular compound isoniazid (INH). So far there is no evidence that the metabolism of INH varies in different strains of mice. It seemed however of some interest to examine the metabolism of this compound and its influence on carbohydrate metabolism in mice compared with Wistar rats, hamsters and *Meriones lybicus* since the biological response appears to differ. At present Dr. Peacock is carrying out biological tests in this laboratory of the possible induction by INH of lung tumours in the laboratory animals mentioned. Among humans there is a quantitative difference in the metabolism of INH and patients have been classified as rapid or slow inactivators depending on the rate at which they metabolise this compound (Evans, Manley and McKusick, 1960).

In 1954, Zamboni and Defranceschi identified the isonicotinoylhydrazones of pyruvic and $\alpha$-oxoglutaric acids in the urine of treated rats. Later Krulik, Simane and Rotter (1962) described the increased clearance of these $\alpha$-oxo acids in the urine of patients after the administration of this drug. In the present experiments an examination has been made of the clearance of pyruvic and $\alpha$-oxoglutaric acids in the urine of mice, *Meriones lybicus*, and rats.

### METHOD

The $\alpha$-oxo acids were estimated as the 2,4-dinitrophenyl-hydrazones by the method of Cavallini and Frontali (1954) in which the hydrazones are separated by paper chromatography.

### *Animals*

The drinking water given to mice contained 0·1 g. INH per 100 ml. while that given to rats and *Meriones lybicus* contained 0·25 g. per 100 ml. In one series of experiments the concentration of INH in drinking water for rats was reduced to 0·1 g. per 100 ml. in order to make a direct comparison with the conditions in the mouse experiments.

The mice, *Meriones lybicus* and rats had been under test for more than one year when the examinations were carried out. When the concentration of $\alpha$-oxo acids in the urine of male mice or rats was compared with the concentration in female urine, no marked differences were detected. Urine samples of $C_3Hf$ and BALB/c strain mice, however, were collected separately.

### RESULTS

An examination has been made of the clearance of pyruvic and $\alpha$-oxoglutaric acids in the urine of mice, *Meriones lybicus*, and rats (Table I). It was found that the concentration of pyruvic and $\alpha$-oxoglutaric acids in the urine of mice and *Meriones lybicus* increased after INH. The level of pyruvic acid in rat urine increased after INH while the $\alpha$-oxoglutaric acid level which was initially relatively high showed a decrease in the treated animals.

TABLE I.—*Concentrations of Pyruvic and $\alpha$-oxoglutaric Acids in Urine after INH*

| Conc. of INH in drinking water per 100 ml. | Mice | | *Meriones lybicus* | | Rats | | |
|---|---|---|---|---|---|---|---|
| | Control | 0·1 g. | Control | 0·25 g. | Control | 0·1 g. | 0·25 g. |
| Number of tests | 5 | 5 | 5 | 7 | 6 | 5 | 5 |
| Pyruvic acid $\mu$g./ml. | 6–31 | 19–80 | 5–33 | 14–78 | 8–17 | 21–99 | 31–102 |
| Average | 14 | 50 | 16 | 50 | 13 | 55 | 70 |
| $\alpha$-oxoglutaric acid, $\mu$g./ml. | 16–47 | 42–200 | 12–36 | 18–66 | 60–117 | 29–70 | 36–67 |
| Average | 26 | 125 | 23 | 47 | 87 | 49 | 46 |

### DISCUSSION

Alterations in the pyruvic acid level have been described in liver failure and other diseases and as the result of administration of chemical agents including isoniazid. In Krulik's cases treatment with INH was followed by increased blood levels of pyruvic and $\alpha$-oxoglutaric acids and there was an increased clearance of these $\alpha$-oxo acids in the urine. Tokutsu (1959) has also described the increased excretion of the $\alpha$-oxo acids in rabbit urine after the administration of the glucuronic acid derivative of INH.

The $\alpha$-oxo acid levels do not always alter in the same way, for example after exercise pyruvic acid is increased while $\alpha$-oxoglutaric acid is unchanged. In the present experiments, in the case of mice the expected increases in pyruvic and $\alpha$-oxoglutaric acid clearances after treatment with INH were found. In the case of the rat, however, while the level of pyruvic acid increased, the level of $\alpha$-oxoglutaric acid decreased. It appears that in this respect carbohydrate metabolism differs in the rat. In addition differences were observed in the untreated animals both in the initial level of $\alpha$-oxoglutaric acid and in the presence of an unidentified hydrazone of high Rf value in the mouse urine extracts which was absent in the rat urine extracts.

Recently there has been much interest in the lactic dehydrogenase (LDH) isoenzymes involved in the conversion of pyruvic to lactic acid in the presence of the coenzyme NAD. Goldman, Kaplan and Hall (1964) found a definite and consistent shift in the pattern of the molecular forms of LDH associated with a large series of malignant neoplasms when compared with benign tumours and normal controls. In 1954 Zatman, Kaplan, Colowick and Ciotti reported the synthesis of the analogue of nicotinamide adenine dinucleotide (NAD) in which nicotinamide is replaced by INH. They suggested that the formation of the analogue may be significant in explaining the antitubercular activity of INH but a more direct effect would be that on pyruvic acid metabolism.

### SUMMARY

Evidence has been obtained of an increased clearance of pyruvic acid in the urine of mice, *Meriones lybicus* and rats treated with INH when compared with the urine of untreated controls. The urinary α-oxoglutaric acid concentration after INH was increased in mice, slightly increased in *Meriones lybicus*, and decreased in rats. In control animals the α-oxoglutaric acid concentration was higher in the rat than in the other two species tested.

I wish to thank Dr. P. R. Peacock for help in preparing the manuscript. Thanks are also due to Mr. C. Fergusson for skilled technical assistance.

### REFERENCES

Biancifiori, C., Ribacchi, R., Bucciarelli, E., DiLeo, F. P. and Milia, U.—(1963) *Lav. Inst. Anat. Univ. Perugia*, **23**, 115.
Cavallini, D. and Frontali, N.—(1954) *Biochim. biophys. Acta*, **13**, 439.
Evans, D. A. P., Manley, K. A. and McKusick, V. A.—(1960) *Brit. med. J.*, ii, 485.
Goldman, R. D., Kaplan, N. O. and Hall, T. C.—(1964) *Cancer Res.*, **24**, 389.
Kulik, R., Simane, Z. and Rotter, Z.—(1962) *Beitr. Klin. Tuberk.*, **125**, 163.
Tokutsu, T.—(1959) *Seikagaku*, **31**, 46.
Zamboni, V. and Defranceschi, A.—(1954) *Biochim. biophys. Acta*, **14**, 430.
Zatman, L. J., Kaplan, N. O., Colowick, S. P. and Ciotti, M. M.—(1954) *J. biol. Chem.*, **209**, 467.